# Biochemical Characterization of *Cassiopea andromeda* (Forsskål, 1775), Another Red Sea Jellyfish in the Western Mediterranean Sea

**DOI:** 10.3390/md19090498

**Published:** 2021-08-31

**Authors:** Gianluca De Rinaldis, Antonella Leone, Stefania De Domenico, Mar Bosch-Belmar, Rasa Slizyte, Giacomo Milisenda, Annalisa Santucci, Clara Albano, Stefano Piraino

**Affiliations:** 1 Institute of Sciences of Food Production (CNR-ISPA, Unit of Lecce), National Research Council, Via Monteroni, 73100 Lecce, Italy; gianluca.derinaldis@ispa.cnr.it (G.D.R.); stefania.dedomenico@ispa.cnr.it (S.D.D.); clara.albano@ispa.cnr.it (C.A.); 2 Department of Biotechnology Chemistry and Pharmacy (DBCF), Università Degli Studi Di Siena, Via A. Moro, 53100 Siena, Italy; annalisa.santucci@unisi.it; 3 Consorzio Nazionale Interuniversitario per le Scienze del Mare (CoNISMa, Local Unit of Lecce), Via Monteroni, 73100 Lecce, Italy; stefano.piraino@unisalento.it; 4 Department of Biological and Environmental Sciences and Technologies (DiSTeBA), Campus Ecotekne, University of Salento, Via Lecce-Monteroni, 73100 Lecce, Italy; 5 Laboratory of Ecology, Department of Earth and Marine Sciences (DiSTeM), University of Palermo, 90128 Palermo, Italy; mar.b.belmar@gmail.com; 6 Department of Fisheries and New Biomarine Industry, SINTEF Ocean, Brattørkaia 17C, 7010 Trondheim, Norway; Rasa.Slizyte@sintef.no; 7 Centro Interdipartimentale della Sicilia, Stazione Zoologica Anton Dohrn, Lungomare Cristoforo Colombo, 90142 Palermo, Italy; giacomo.milisenda@szn.it

**Keywords:** jellyfish, antioxidant activity, bioactive marine compounds, zooxanthellate jellyfish, antioxidants, fatty acids, collagen, nutraceuticals, alien species

## Abstract

Increasing frequency of native jellyfish proliferations and massive appearance of non-indigenous jellyfish species recently concur to impact Mediterranean coastal ecosystems and human activities at sea. Nonetheless, jellyfish biomass may represent an exploitable novel resource to coastal communities, with reference to its potential use in the pharmaceutical, nutritional, and nutraceutical Blue Growth sectors. The zooxanthellate jellyfish *Cassiopea andromeda*, Forsskål, 1775 (Cnidaria, Rhizostomeae) entered the Levant Sea through the Suez Canal and spread towards the Western Mediterranean to reach Malta, Tunisia, and recently also the Italian coasts. Here we report on the biochemical characterization and antioxidant activity of *C. andromeda* specimens with a discussion on their relative biological activities. The biochemical characterization of the aqueous (PBS) and hydroalcoholic (80% ethanol) soluble components of *C. andromeda* were performed for whole jellyfish, as well as separately for umbrella and oral arms. The insoluble components were hydrolyzed by sequential enzymatic digestion with pepsin and collagenase. The composition and antioxidant activity of the insoluble and enzymatically digestible fractions were not affected by the pre-extraction types, resulting into collagen- and non-collagen-derived peptides with antioxidant activity. Both soluble compounds and hydrolyzed fractions were characterized for the content of proteins, phenolic compounds, and lipids. The presence of compounds coming from the endosymbiont zooxanthellae was also detected. The notable yield and the considerable antioxidant activity detected make this species worthy of further study for its potential biotechnological sustainable exploitation.

## 1. Introduction

The benthic scyphozoan *Cassiopea andromeda* (Cnidaria, Rhizostomeae) (Forsskål, 1775), also known as the upside-down jellyfish [1,2], is native of the Red Sea. Nearly 34 years after the opening of the Suez Canal (dated 1867), it entered the Eastern Mediterranean Sea as a non-indigenous species [3]. The first records of *Cassiopea andromeda* species from the central Mediterranean Sea were in Malta harbor [4] and in Tunisia [5]. In 2014, *Cassiopea andromeda* was suddenly detected in Italian waters, spotted in sheltered eutrophic waters, such as the harbor of Augusta (Sicily) (Domenico Catalano, personal communication), the small marina of Acquasanta, near Palermo (Tony Scontrino, personal communication) and [6] and in the inner marina La Cala [7]. In 2017 it was first spotted in the eutrophic waters of Mar Menor, Spain [8]. Now, small blooms are occurring almost every year in the Palermo harbor (personal observations). The upside-down jellyfish found into the Palermo harbor was identified by COI molecular barcoding as *Cassiopea andromeda* [9]. 

The genus *Cassiopea* includes a group of stationary jellyfish species usually associated with shallow water, living in tropical and sub-tropical areas characterized by mangrove habitats, laying with its bell on the sea floor and its oral arms directed upwards to the water surface. It currently includes nine molecular recognized species even though other species are still considered by morphological analysis only [10,11,12]. The peculiar body posture of *C. andromeda* and its congeneric species has a trophic reason. As other jellyfish species, *Cassiopea spp.* host endosymbiotic microalgae (dinoflagellates, family Symbiodiniaceae), called zooxanthellae in their body tissues [13]. *Cladocopium spp.*, often associated with *Symbiodinium* and *Breviolum spp.*, are the most common endosymbionts of *Cassiopea* [14,15,16].

The seasonal blooms of scyphozoan jellyfish (JF) in the Mediterranean represent a source of unexploited biomass: conversely, the use of Asiatic JF as human food and for their pharmaceutical proprieties in Eastern countries dates back over a thousand years [17]. The market value of this kind of commodity is indeed considerable but, in Europe, there is yet no tradition of the food use of jellyfish, no food market related to JF as food, or any other use. Only recently, the Mediterranean species *Rhizostoma pulmo* was suggested as raw material for human foods [18,19,20]; however, jellyfish living in EU seas are currently labelled as “novel food” in European Regulation and its consume is still not allowed (EU Regulation 2015/2283 of 25/11/2015 http://eur-lex.europa.eu/eli/reg/2015/2283/oj accessed on 22 April 2021). 

Other studies on Mediterranean outbreak-forming jellyfish species [18,21,22] showed that the jellyfish outbreaks could turn out to be a source of value-added healthy food and a potential source of compounds for nutraceutical, cosmeceutical, or pharmaceutical applications. Marine bioprospecting of new natural products has yielded several thousand novel molecules in the last decades, and it is expected that research in this field will lead to the discovery of numerous new marine natural products with high bioactivity [23,24,25]. Sessile cnidarians, such as sea anemones and corals, are regarded as key organisms for bioprospecting [26,27]. Cnidarian jellyfish, with their large seasonal biomass outbreaks and their unequalled developmental potential in the animal kingdom [28], currently represent an issue in many coastal areas [29] but they could also represent a valuable source of protein and bioactive compounds [21,22,30].

Overall, jellyfish represent a relatively untapped natural resource, with a limited number of derived products [24,31,32,33,34,35,36,37,38]. Previous studies mostly focused on jellyfish proteinaceous compounds, yet other non-proteinaceous components may have high bioactivity potential, as the new polysaccharide (JSP-11) from *Rhopilema esculentum* stimulating a macrophage-mediated immune response in mice via several signalling pathways [39]. Scyphomedusae, indeed, are made of water for more than 95% [40], while the jellyfish dry mass is composed mainly by salt and proteins, and by other non-proteinaceous compounds (carbohydrates, phenolic compounds and lipids) that are minor components. However, the percentage of these compounds can be very different among jellyfish, with taxa in the order Rhizostomeae containing more proteins (mainly collagen) than any other scyphomedusae [21,41].

Currently, marine collagen is considered a good alternative to the classical terrestrial source of collagen [42], with no risk to transfer of bovine spongiform encephalopathy (BSE). Collagen accounts for up to 30% of mammal proteins [43] and for up to 60% of proteins in jellyfish [44,45]. Between marine organisms, the yield of collagen obtained from jellyfish is usually greater [41], with the pepsin solubilization that seems the most effective extraction process [21,46]. Jellyfish collagen seems highly biocompatible for human body [47] and it seems to have similarity with mammalian type I collagen [45]. Moreover, collagen and collagen hydrolysate (collagen-derived peptides) have been shown to exert several immunomodulatory, antioxidant, photoprotective, and wound healing effects [48,49,50]. Jellyfish are very poor in lipids; however, several species benefit from their symbiotic association with intracellular dinoflagellate microalgae, also known as zooxanthellae [16] characterized by high concentrations of lipids, carotenoids, phenolic compounds and photosyntetic pigments. *Cotylorhiza tuberculata* jellyfish, which hosts the symbiotic dinoflagellate *Symbiodinium microadriaticum*, is known to possess higher amount of lipids than the asymbiotic *Rhizostoma pulmo* and *Aurelia coerulea* jellyfish [21]. Scant information is also available on phenolic compounds in jellyfish [18,21], besides the zooxanthellate *C. tuberculata* [22].

Here we report the biochemical composition and antioxidant activity of jellyfish *Cassiopeia andromeda* collected in autumn 2017, from “La Cala” marina, in the harbor of Palermo. We set up a method for the differential extraction of soluble and insoluble components of jellyfish biomass. We evaluated protein, phenolic and lipid contents, and aminoacids and fatty acids composition from whole jellyfish or from different body parts (umbrella and oral arms), and the corresponding antioxidant activities of aqueous and hydroalcoholic extracts and insoluble fractions.

## 2. Results and Discussion

### 2.1. Jellyfish Biometric Data

Biometric data (Table 1) revealed that the investigated jellyfish specimens were almost homogeneous in size, with the umbrella (UMB) diameter ranging 13.5–17.5 cm, with a mean of 15.0 ± 1.3 cm, and the fresh weight (FW) of the whole specimen ranging 152.4–296.5 g, with a mean of 233.1 ± 46.1 g. 

The resulting total dry weight (DW) of the jellyfish ranged from 11.6 g to 22.4 g, with a mean value of 17.4 ± 3.1 g. As a consequence, the yield in dried biomass, referred to the corresponding fresh weight (Yield %FW), ranging 6.9–8.1%, with the mean value of 7.5% (Table 1). This yield is high as compared to other jellyfish, ranging 2.2–3% in *Aurelia sp*, and 4.1–6.8% in *Rhizostoma pulmo*, whereas yield ranging 1.1–10.5% in *Periphylla periphylla* [51], a rhizostomid zooxanthellate jellyfish, where the large range is maybe due to the different content in microalgae [21,44,48,51,52]. The yield data refer to specimens with a diameter between 13 cm and 17 cm; yet, according to local physic-chemical conditions and organic matter availability, *C. andromeda* can grow up to 49 cm of diameter [53]. In *C. tuberculata*, the DW/FW ratio rise in proportion with the size of the specimens [21], therefore this may be also expected to be the case for large *C. andromeda* specimens. Interestingly, the mean size of *C. andromeda* jellyfish occurring in the shrimp’s farms in the Brazilian coasts appears three times larger than the natural population living in the mangrove; apparently, this is not dependent on the overall nutrient concentrations (higher in the mangroves than in the shrimp’s farms) but, most probably, to the physic-chemical high stability in the shrimp’s farm, which enhances jellyfish survival and, eventually, growth [53], more than in the seasonally highly fluctuating conditions of the natural mangrove habitats.

### 2.2. Jellyfish Biomass Composition

The biomass characterization was firstly based on the analysis of the amino acid and fatty acid composition of the whole jellyfish. Then, the extraction of water-soluble and hydroalcoholic-soluble compounds and the following sequential enzymatic hydrolysis of the insoluble compounds (mainly composed of proteins) were carried out. Both soluble and insoluble compounds were analyzed for contents of protein and phenolic compounds, and for antioxidant activity.

#### 2.2.1. Amino Acid Composition of Whole *C. andromeda* Jellyfish

The amino acid composition of *C. andromeda* jellyfish is reported as percentage per dry weight of jellyfish (%DW) and as percentage of total amino acids (Table 2). The most abundant amino acids were Glutamic acid +Glutamine (Glx) and Glycine (1.80 ± 0.03 and 1.72 ± 0.12 g/100 g of DW, respectively); the latter, in general, spaced in every third residue of the collagen molecules, except the first 10 amino acids at C-terminus region and the last 14 amino acids at N-terminus region [54], and accounts for about one-third of total residues in jellyfish *Rhopilema esculentum* collagen [55]. 

Aspartate + Asparagine (Asx 1.29 ± 0.07 g/100 g of DW), Lysine (1.06 ± 0.01 g/100 g of DW), and Arginine (1.02 ± 0.02 g/100 g of DW,) were also found at high content. The imino acids Proline (0.97 ± 0.02 g/100 g of DW) and Hydroxyproline (0.26 ± 0.02 g/100 g of DW) are also abundant (6.04% and 1.64% of total amino acid, respectively) in collagen, enhancing the stability of the triple helix by a network of hydrogen bonds formed by bridging water molecules and the pyrrolidine rings of these imino acids [56,57]. Alanine and Taurine (both 0.96 ± 0.02 g/100 g of DW) content in *C. andromeda* was also found to be high. Cystine, the dimer of Cysteine, was also found (0.28 ± 0.07 g/100 g of DW) in the reduced form. The remaining amino acids (Threonine, Serine, Valine, Methionine, Isoleucine, Leucine, Tyrosine, Phenylalanine, Histidine) ranged from 0.2 to 0.8 g/100 g of DW while Hydroxylysine and Tryptophan as well as Methionine sulfoxide were not detected. Except Tryptophan, all essential amino acids—Histidine, Isoleucine (Ile), Leucine (Leu), Lysine (Lys), Methionine (Met), Phenylalanine (Phe), Threonine (Thr), and Valine (Val)—were present. Tryptophan was not detected most probably due to degradation during the pretreatment of sample prior analysis of amino acids.

The total amount of amino acids was 15.68 ± 0.09 g/100 g of whole lyophilized jellyfish *C. andromeda* more than twice time the amount of some *Rhizostoma pulmo* and *Pelagia noctiluca* samples (6.1 ± 0.09 g/100 g and 8.1 ± 0.3 g/100 g, respectively, of lyophilized whole jellyfish), which were analyzed in parallel. 

#### 2.2.2. Jellyfish Soluble Compounds

**Figure 1:**
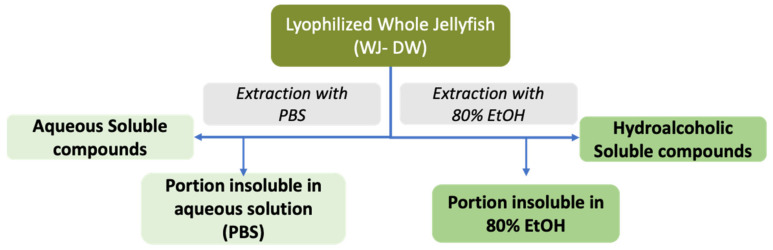
Scheme of the two experimental approaches, for the extraction of water- and hydroalcoholic-soluble compounds from *C. andromeda* jellyfish samples.

To establish the presence of extractable compounds and their chemical nature, two solvent systems, Phosphate-Buffered Saline (PBS) and 80% ethanol solution (80% EtOH), were used to solubilize water-soluble compounds and hydrophobic compounds, respectively [21,22] as showed in Figure 1. 

A first analysis was performed on the whole jellyfish (WJ) biomass. In order to verify the extraction efficiency of the two solvent systems (PBS and 80% EtOH), the extraction yield (% DW) was estimated in terms of amount (g) of dried extract compared to the dry weight of the lyophilized whole jellyfish, for each solvent system (Table 3).

Data in Table 3 shows that a considerable amount of dry extract was recovered by both the solvent systems: the saline solution (PBS) yields 83.4% on DW (*w*/*w*) bases, while ethanol solution was able to extract 42.5%. The yields in term of fresh weight (Yield %FW) was theoretically calculated by considering the mean value of 7.5% on FW showed in Table 1: this value was 7.1% and 3.2% for PBS and 80% EtOH extracts, respectively. The data demonstrated that extraction from dried biomass of *C. andromeda* in the aqueous solution (PBS) allows a higher yield of compounds than extraction in hydroalcoholic solution (80% EtOH). The data related to the hydroalcoholic extraction yield found in *Cotylorhiza tuberculata*, another zooxanthellate jellyfish—in the same experimental condition used in this study—provided a comparable extraction yield of 43.6 ± 4.1% of DW (*w*/*w*), and 11.7 ± 1.7% of FW (*w*/*w*) [22], indicating similarity in term of yield, independently from the species and the size of the jellyfish. To our best knowledge, no data are available so far on PBS or other saline solution extractions relative to jellyfish-related compounds.

#### 2.2.3. Jellyfish Lipid Composition 

The lipid analysis was carried out on the whole jellyfish samples and only on the hydroalcoholic extract as the water-soluble extract obviously does not contain conventional lipids. To characterize the lipid content and fatty acid composition of *C. andromeda* jellyfish, the total lipids of the whole jellyfish as well as of the hydroalcoholic extract were extracted and analyzed. Total lipids extracted by chloroform/methanol from the whole lyophilized jellyfish (WJ) and from the dried 80% EtOH extract are shown in Table 4. A considerable amount of lipids was obtained from the whole *C. andromeda* tissue, about 9.4 ± 0.4 mg/g of lyophilized jellyfish samples, corresponding to about 1% of the DW. The extraction by 80% EtOH was quite efficient being able to extract an amount of total lipids equal to 6.2 ± 0.5 mg/g of lyophilized jellyfish (0.62% of DW) and corresponding to 15.5 ± 0.5 mg per gram of hydroalcoholic extract. The theoretically calculated percentage of total lipids with respect to fresh weight of jellyfish is approximately 0.07% (0.07 g/100 g FW) of which about 0.05% (*w*/*w*) of the FW were extractable by hydroalcoholic extraction.

The fatty acid (FA) composition of both the whole jellyfish tissues (WJ) and hydroalcoholic soluble compounds extracted by 80% ethanol from *C. andromeda* is shown in Table 5 and was expressed as percentage of the total FA. The saturated fatty acids (SFA) and polyunsaturated fatty acids (PUFA) were the most representative FA in the whole jellyfish (WJ), being approximately 48% (SFA) and 44% (PUFA), respectively, while monounsaturated fatty acids (MUFA) represented only the 8% of total FA. The extraction with 80% ethanol seems to favor the extraction of PUFA, whose value (about 63%) was higher than SFA (about 31%), while the MUFA always represented the minor part (6%).

The most represented SFAs in the whole jellyfish were palmitic (C16:0, 21.2%), stearic (C18:0, 12.5%) and lauric (C12:0, 9.3%) acids, whose values are higher than in the hydroalcoholic extract where they were 13.9%, 9.9%, and 1.5%, respectively. The SFAs myristic (C14:0, 4–5%) and arachidonic acid (C20:0, 0.6%) acids were represented in the same negligible percentage in both samples.

The 80% EtOH extract resulted enriched in PUFAs as compared to the whole jellyfish and the composition of PUFAs showed higher percentage of almost all the extracted fatty acids in the 80% EtOH extract than in the whole jellyfish sample. Arachidonic (C20:4) and docosahexaenoic (C22:6, DHA) acids were 14.2% and 11.0%, respectively, in the total JF tissues and 19.2% and 17.8% in the hydroalcoholic extract, respectively. In the hydroalcoholic extract linoleic acid (C18:2, 1.9%), docosatetraenoic acid (C22:4, 4.2%) and docosapentaenoic acid (C22:5, DPA, 5.1%) roughly doubled those found in the WJ samples (1.9%, 4.2%, 5.1%, respectively). Instead, linolenic acid (C18:3, ALA) and eicosapentaenoic acid (C20:5) values were only slightly higher in hydroalcoholic extract (3.2 and 3.5% as compared to 2.6 and 2.1%), in addition the isomeric form of linoleic acid, isolinoleic acid (C18:2 *cis-6,9*), was only detected in the whole jellyfish. Notably the stearidonic acid (C18:4, SDA), a novel omega-3 PUFA that has generated recent interest [58], was also found. In general terms, this vegetal-derived PUFA, is the immediate product of the metabolic conversion of linolenic acid (ALA) catalyzed by delta-6 desaturase. This step, however, is not effective in humans, and this is the reason why dietary ALA is poorly converted to longer carbons-chains ω3-PUFA (EPA, DPA, and DHA) and the consumption of oils rich in SDA can result in an enrichment of tissues with EPA, DPA, or DHA [59]. SDA is a minor constituent of many fish oils but it is also found in the seeds of a number of plants. It is easy to presume its origin, as well as the origin of most of PUFA from the symbiotic *Symbiodiniaceae* dinoflagellates present in *C. andromeda* tissues.

The composition in MUFA of the whole jellyfish and the hydroalcoholic extract was similar, with the finding of comparable level of palmitoleic acid (C16:1, about 4%), oleic acid (C18:1, about 2.5%), and isooleic acid (C18:1 *trans-10*, 0.5%).

From a nutritional and nutraceutical point of view however, in all the two samples the ω6/ω3 ratio did not change and was less than 1 (ω6/ω3 0.7, Table 5) thus highlighting a greater presence of ω3-FAs in *C. andromeda* tissues than ω6-FAs. In western country diets the ω6/ω3 ratio is around 15–17 [60], while several studies attest that its reduction to 2.5–5 values has beneficial and preventive effects against the most common “diseases of well-being”. The daily consumption of the dietary omega-3 PUFA (ALA, EPA, DPA, and DHA nowadays considered essential fatty acids) is recommended in several Countries by health organizations and governmental agencies. The molecular mechanisms by which PUFA affect the human health, is linked to the enrichment of cell membranes with omega-3 PUFA characterized by long-chain 20-carbons (EPA) and 22-carbons (DHA), impacting thus trans-membranes protein functions, cell signaling, and genes expression [59]. These changes are recognized to have health benefits in humans, especially relating to cardiovascular diseases [61], breast cancer [62], inflammation in patients with rheumatoid arthritis and asthma [63]. 

#### 2.2.4. Soluble Biomass Distribution in Umbrella and Oral Arms

Different body parts, namely the Umbrella (UMB) and the Oral Arms (OA) excised from different specimens of *C. andromeda* were separately analyzed in order to estimate the yield of the two jellyfish body parts. Table 6 shows the average yields of the extracts by using PBS or 80% EtOH, (Extract DW, g) of UMB and OA. In both, UMB and OA, the PBS extraction yield was higher than that with ethanol solution. The percentage yields for PBS extraction were 94.5 ± 0.02% and 95.8 ± 0.01% of the lyophilized umbrellas and oral arms, respectively, while the percentage yields for hydroalcoholic extraction were 41.6 ± 0.03% and 43.5 ± 0.01%, respectively.

No differences between UMB and OA samples in terms of yield were found. Given that the yields of both aqueous and hydroalcoholic extracts from the whole jellyfish were quite high, ranging approximately from 50 to 95% of DW (Table 3), it appears that the extractable compounds are equally distributed in the UMB and OA body parts (Table 6). 

Generally, jellyfish body parts have a different composition due to their diverse role. In pelagic cnidarians, umbrellas have well developed muscle cell organization, useful for active contraction and movement, and so the bell pulsations are relied on as their mode of locomotion [64,65,66,67], while oral arms and tentacles are typically used for capturing prey [68,69].

A different case is represented by the upside-down jellyfish *Cassiopea*, which have a less active locomotion being more similar to sessile organisms. The contractile nature of the umbrella and oral arms in *Cassiopea andromeda* specie was studied by mathematical models and fluid dynamics [70,71]. The oral arms seem to have active contractile capacity and a particular structure that make them different from the manubrium of many cnidarian jellyfish. The absence of a primary mouth is balanced by multiple microscopic, secondary mouth-like openings, used for a microphagous mode of feeding; also, the oral arms are filled by secretory cells producing an abundant secretion of defensive mucus. Apparently, heterotrophic feeding seems more important than the symbiotic dinoflagellate-derived photoautotrophy (mainly as a source of lipids) [14,53,72].

#### 2.2.5. Partial Characterization of Soluble Compounds in Umbrella and Oral Arms of *Cassiopea andromeda*

**Figure 2:**
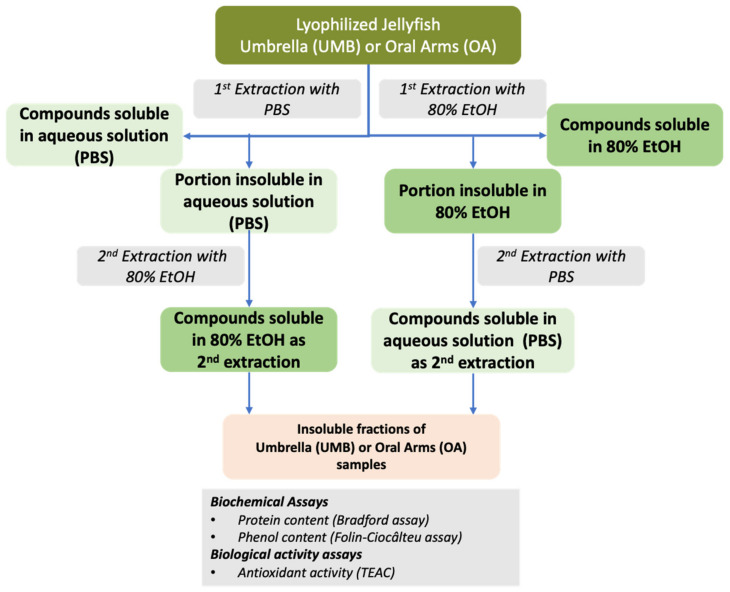
Scheme of the experimental approach used to evaluate the extraction efficiency of soluble compounds by double alternated extractions with PBS and 80% ethanol (EtOH).

The PBS and 80% ethanol extractions of soluble compounds from *Cassiopea andromeda* UMB and OA samples were further defined in order to get additional information about the extraction strategy. The scheme in Figure 2 shows the procedure based on single and double extractions used to evaluate the extraction efficiency of different classes of molecules, such as proteins and phenolic compounds, from jellyfish tissues. The protein and phenolic compounds’ contents as well as the antioxidant activity (AA) were evaluated in both aqueous (PBS) and hydroalcoholic (80% EtOH) extracts of both umbrellas and oral arms in the first and second extractions. 

##### Soluble Protein and Phenolic Compound Content in Aqueous and Hydroalcoholic Extracts

**Figure 3:**
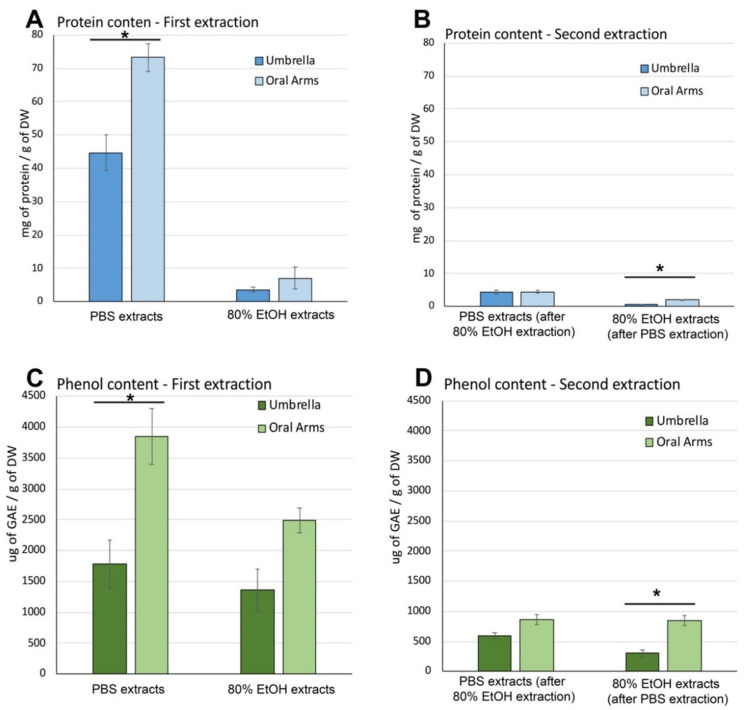
Content of proteins (**A**,**B**) and phenolic compounds (**C**,**D**) in PBS and 80% EtOH extracts from umbrella and oral arm samples of *Cassiopea andromeda*. A and C: First extraction with PBS and 80%EtOH; B and D: Second extraction carried out with different solutions (PBS extraction after 80% EtOH extraction and 80% EtOH after PBS extraction). Data of protein content are expressed as mg of proteins per gram of dry weight (DW); Phenol content is expressed as µg of Gallic Acid Equivalent (GAE) per gram of DW. Data are mean (*n* = 6), of three independent experiments bars represent ± standard deviation (SD); * *p* < 0.05.

Figure 3 shows the protein and phenolic content and antioxidant activity of both the first and second extraction, with PBS and 80% ethanol, in umbrellas and oral arms. The total amount of soluble proteins extracted by PBS and 80% EtOH (Figure 3A) was evaluated by Bradford assay. PBS extraction performed as first extraction was more efficient than 80% EtOH since was able to extract 44.72 ± 3.10 mg of proteins per g of DW of umbrella samples and 73.21 ± 5.90 mg/g DW from oral arms as compared to hydroalcoholic extracts, which showed a protein content of 3.55 ± 0.68 mg/g of DW and 7.03 ± 3.37 mg/g DW from UMB and OA, respectively. Furthermore, a significant difference in protein content was evident between umbrella and oral arms in PBS extracts, while no difference was found in 80% EtOH extracts (Figure 3A, First extraction).

A second extraction of soluble compounds was performed in order to optimize the extraction process and verify the effect of multiple extractions (Figure 3B,D). At this aim, the insoluble residues obtained from the first extraction in PBS were subject to another extraction step, using the 80%EtOH solution, as well as the insoluble residues obtained from the first extraction in 80%EtOH were subjected to PBS extraction as second extraction (Figure 2). 

The protein content in the PBS extract obtained from residues after the first hydroalcoholic extraction, were 4.33 ± 0.67 mg and 4.42 ± 0.39 mg of proteins/g of DW in umbrella and oral arms, respectively (Figure 3B, Second extraction). The values were very low as compared to both the PBS and 80% EtOH extractions, performed as first extraction. Similarly, the second extractions with 80% EtOH, carried out after the PBS extraction, provided a very low amount of protein, indeed 0.60 ± 0.02 mg and 1.90 ± 0.68 mg/g of DW in extracts from umbrella and oral arms, respectively were found. No difference between protein content in umbrella and oral arms was detected for both extraction systems (Figure 3B).

Actually, the pre-extraction with 80% EtOH prevents the protein extraction by PBS, maybe due to the protein denaturation and possible precipitation of the proteins in insoluble forms due to the 80% ethanol solution [73]. Therefore, PBS extraction results the most efficient solution to extract soluble compounds from *C. andromeda* tissues, while the 80% EtOH extraction system, used both directly and as secondary step, provides a low, although more specific and selective, protein yield [74].

The content of total phenolic compounds was evaluated as micrograms of gallic acid equivalent (GAE) per g of jellyfish dry weight (μg GAE/g DW) and was measured in all the four extracts (Figure 3C,D) obtained from both UMB and OA of *C. andromeda*. As shown in Figure 3C, the first extraction in PBS was able to extract about 1785 ± 380 µg of GAE/g of DW from umbrellas and about 3851 ± 450 µg of GAE/g of DW from oral arms, while the 80% EtOH solution was able to extract 1358 ± 340 GAE/g of DW from umbrellas and 2483 ± 201 GAE/g of DW from oral arms. This confirmed that a difference between UMB and OA in extractable compounds is evident also for phenolic compounds. In addition, statistical analysis indicates no significant difference between extractable phenolic compounds by PBS and ethanol solutions from umbrella, while a significantly higher amount of PBS extractable phenolic compounds was detected in oral arms as compared to 80% EtOH extraction solution.

The total contents of phenolic compounds obtained from the second extractions are shown in Figure 3D. From one third to one sixth of the quantity presents in the first extraction solution was still extractable in the subsequent extraction. No difference was detected for extractable phenolic compounds in OA with both PBS after EtOH extraction and with 80% EtOH after PBS extraction, while significant less phenolic compounds were extracted in 80%EtOH after PBS extraction in umbrella samples (Figure 3D).

##### Antioxidant Activity in Aqueous and Hydroalcoholic Extracts

**Figure 4:**
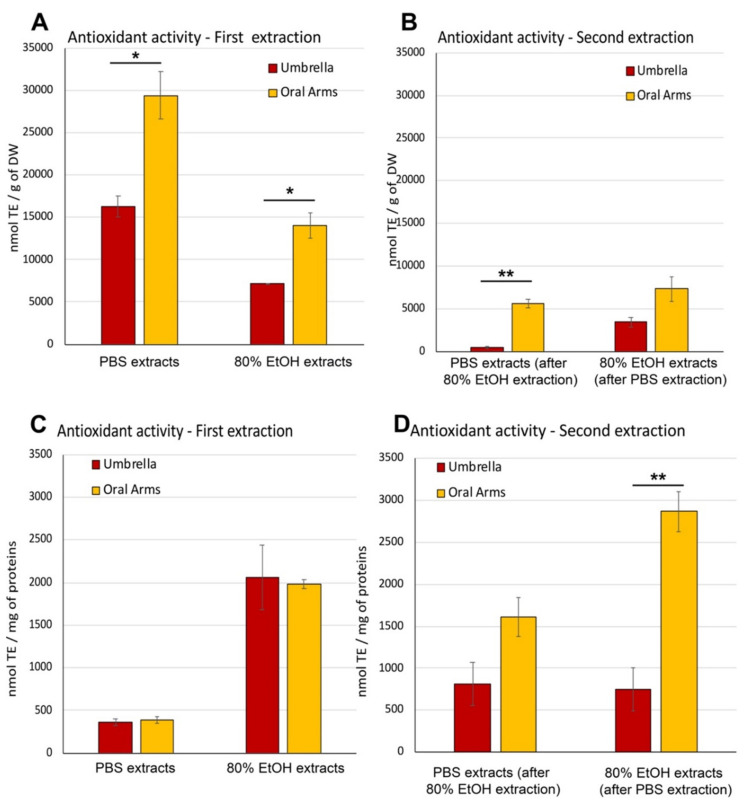
Antioxidant activity in PBS and 80%EtOH extracts from umbrella and oral arm samples of *Cassiopea andromeda*. (**A**,**C**) First extraction; (**B**,**D**): Second extraction. The first and second extraction were carried out with different and alternate solutions (PBS followed by 80% EtOH or 80%EtOH followed by PBS). Data of antioxidant activity are expressed as Trolox equivalent per gram of DW (nmol of TE/g of DW) in A and B, and expressed as Trolox equivalent per mg of proteins (nmol of TE/mg of proteins) in C and D. Data are mean (*n* = 6), bars represent ± standard deviation (SD); * *p* < 0.05, ** *p* < 0.01.

High antioxidant activity (AA) was detected in all jellyfish extracts of both aqueous- and hydroalcoholic-soluble compounds obtained from *C. andromeda*. In Figure 4 the AA measured in the four different extracts and expressed as nmol of TE/g of DW and as nmol of TE/mg of proteins are shown in A, B and in C,D, respectively. The AA expressed as nmol Trolox equivalent per gram of DW (Figure 4A,B), evaluated in the first extraction samples were higher than that in the second extraction samples for all the solvent systems. In addition, the AA measured in the extracts of OA was higher than in those of the UMB samples. When comparing the two solvent systems of the first extraction, the 80% EtOH extracts resulted in higher AA than the PBS extracts. Indeed, PBS extracts from UMB and OA were 16291 ± 1250 nmol of TE/g of DW and 29381 ± 2789 nmol of TE/g of DW, respectively, and it was significantly higher than in the 80% ethanol extracts of UMB and OA (7177 ± 55 and 13979 ± 1500 nmol of TE/g of DW, respectively). The trend was similar in the second extractions (Figure 4B) except than for the extracted amounts: PBS was 5–10 times less efficient when following the 80% EtOH procedure, while the 80% EtOH, used as second extraction solvent system after PBS extraction, resulted nearly 0.5 times lower than the AA activity measured in 80% EtOH as first extraction.

In order to consider the different extraction capacity and selectivity of the used solvents and the different compounds with AA present in the samples (umbrellas and oral arms), the antioxidant activity was expressed also as nmol Trolox equivalent per mg of proteins (nmol TE/mg of proteins). The normalized data can indirectly express the qualitative differences among samples related to their antioxidant capability. No differences in the AA between umbrella and oral arms samples were found in both extraction systems (PBS and 80% EtOH) (Figure 4C,D). Overall, the AA measured in the first extractions carried out in 80% EtOH was significantly higher than in PBS extracts regardless of the type of sample (UMB or OA), indicating that the hydroalcoholic extraction is selective for compounds with high antioxidant activity (Figure 4C).

The antioxidant activity measured per mg of proteins contained in the second extractions (Figure 4D) confirmed that PBS extracts, from both UMB and OA, and 80% EtOH from OA resulted in higher AA values than in the first extraction, indicating that the AA was not only exerted by the proteinaceous components. No significant differences were found between the AA in UMB and OA samples (815 ± 257 and 1610 ± 235 nmol TE/mg of proteins, respectively), extracted by PBS while a significantly higher AA was detected in OA as compared to UMB extracted by 80% EtOH after PBS extraction (Figure 4D). 

#### 2.2.6. Umbrella and Oral Arms Insoluble Biomass Characterization

**Figure 5:**
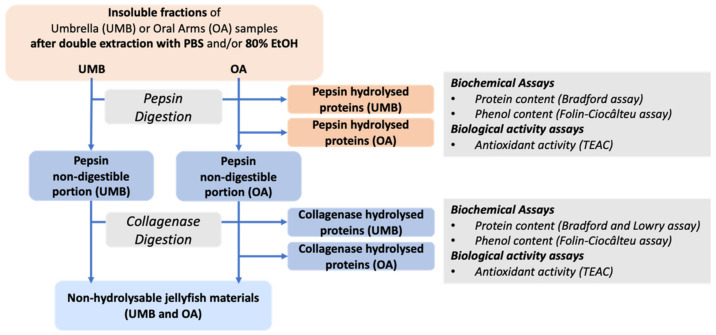
Scheme of the experimental approach used to characterize the insoluble biomass of *C. andromeda* jellyfish. Samples of umbrellas (UMB) and oral arms (OA) after the extractions of soluble compounds by PBS and 80% EtOH, are subjected to enzymatic digestion as indicated in the scheme.

In order to characterize the insoluble fraction resulting after the double extractions in aqueous (PBS) and hydroalcoholic (80% EtOH) mainly consisting of proteinaceous material [21,48], both UMB and OA insoluble fractions were subjected to sequential enzymatic hydrolysis. A digestion with pepsin followed by a second digestion with collagenase on pepsin-undigested fraction was performed (Figure 5). Pepsin breaks down a wide range of proteinaceous compounds [75], its optimal temperature is 37 °C; however, in order to preserve possible sample bioactivity, it has been used at 4 °C for a longer time following the protocol set up by Leone et al. [21]. In addition, pepsin is able to cleave the non-triple collagen domains without affecting the triple helix [76]. The pepsin-undigested proteins, mainly consisting of the triple helix domains of collagen, were then hydrolyzed by bacterial collagenase [21]. The sequential process was preferred instead of a combined enzymatic hydrolysis in order to have a better control of the digestion parameters and the resulting peptide composition, as well as a quantitative evaluation of the content of collagen and non-collagen proteins [77].

**Figure 6:**
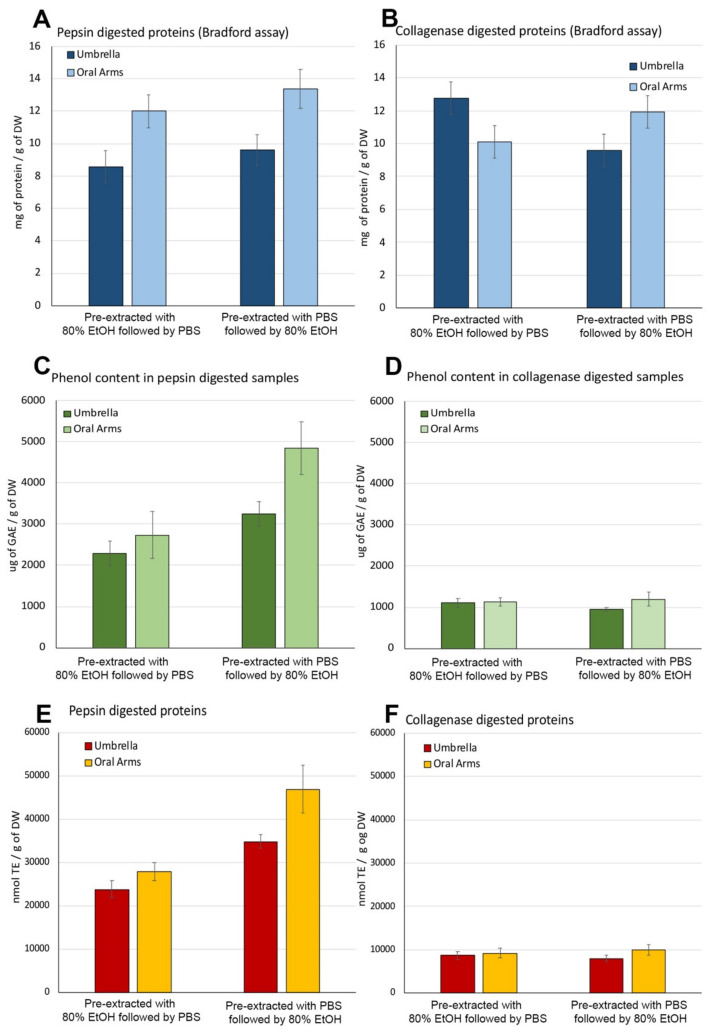
Protein content (**A**,**B**), phenolic compounds’ content (**C**,**D**) and antioxidant activity (**E**,**F**) measured in the supernatants resulting from sequential hydrolysis of the insoluble compounds hydrolyzed with pepsin (**A**,**C**,**E**) followed by collagenase (**B**,**D**,**F**) in samples of umbrella and oral arms of *Cassiopea andromeda*, pre-extracted with PBS followed by 80% EtOH or with 80% EtOH followed by PBS. Protein content is expressed as mg per gram of dry weight (DW), total phenolic content is expressed as µg of gallic acid equivalent (GAE) per g of DW and antioxidant activity is expressed as nmol of Trolox eq. (TE) per g of DW. Data are the mean of three independent experiments performed in triplicate; data are means with ± standard deviation (SD).

The total protein and phenol contents as well as the antioxidant activity measured in all samples digested by pepsin followed by collagenase are reported as follows (Figure 6). 

##### Protein Content in Pepsin- and Collagenase-Hydrolyzed Jellyfish Fractions

Pepsin digestion was performed on both the aqueous and hydroalcoholic insoluble compounds obtained after the two extraction steps in PBS and 80% EtOH (Figure 5), on both UMB and OA samples. The protein content in the supernatant after pepsin digestion measured in OA samples was higher than those in UMB samples (Figure 6A), regardless of the type of previous extraction (PBS followed by 80% EtOH, or 80% EtOH followed by PBS), demonstrating that the type of extraction previously carried out did not change the yield of proteins digestible by pepsin. Indeed, the amount of pepsin-hydrolysates from the UMB was 8.6 ± 1.0 mg after PBS followed by 80%EtOH and 9.61 ± 0.95 mg after 80% EtOH followed by PBS per gram of DW, while hydrolysates concentration from OA, was 12.0 ± 1.0 mg and 13.4 ± 1.2 mg/g of DW after PBS followed by 80% EtOH and 80% EtOH followed by PBS extraction, respectively (Figure 6A).

The insoluble pellet not digested by the endopeptidase pepsin is likely mainly composed of insoluble triple helix collagen. In order to evaluate the amount of collagen in UMB and OA of *C. andromeda*, the residual biomass after pepsin-digestion was subjected to enzymatic hydrolysis with collagenase, a bacterial endopeptidase able to hydrolyze the triple-helix of collagen (fibrillar collagen), producing smaller peptides [21,48]. Figure 6B shows the amounts of collagenase-hydrolyzed proteins evaluated by Bradford assay [78] was about 12.8 ± 0.3 and 10.1 ± 0.3 mg/g of DW in umbrella and oral arm samples respectively, pre-extracted with 80%EtOH followed by PBS. A similar amount of fibrillar collagen, namely 9.6 ± 0.1 and 11.9 ± 0.5 mg/g of DW was measured in samples of umbrella and oral arm, respectively, previously extracted by PBS and then with the hydroalcoholic solution. No significant differences between umbrella and oral arms as well as between pre-extraction systems were found.

Protein quantification was performed on collagenase-digested samples by Bradford assay to ensure compliance among protein measurement data in the diverse fractions. Anyway, Bradford assay including its improved version [79], is not recommended assay for collagen analysis due to specific aminoacidic composition of collagen and to the action mechanism of the dye. In order to estimate more accurately the collagen content in our samples, a simple and sensitive Lowry’s method, first developed by [80] and then slightly modified by [81], was used. 

The values of protein content in collagenase-hydrolyzed samples evaluated by the modified Lowry method were found three times higher than the values resulting from Bradford assay. Collagen concentration was about 32.6 ± 1.5 mg and 34.6 ± 1.0 mg/g of DW in umbrella and oral arm samples, respectively, when pre-extracted with 80% EtOH followed by PBS. In UMB and OA samples pre-extracted with PBS followed by 80% EtOH, collagen amounts were about 22.0 ± 1.1 mg of DW for UMB, and 29.8 ± 0.5 mg/g of DW for OA. Even by measuring the collagen content with Lowry’s assay, no difference between UMB and OA as well as between pre-extraction protocols was found. 

The total collagen content in the whole *C. andromeda* jellyfish, estimated by the more accurate HPLC quantification of hydroxyproline, indicated a value of 20.8 ± 0.2 mg/g of DW of total collagen, as indicated by Khong et al. [44].

Depending on the used assays, the amount of total collagen in samples of *C. andromeda* seems to range from 22 to 60 mg/g of DW, being the first value more similar to the value calculated by hydroxyproline quantification method. 

These values are also in agreement with the hypothesis that pepsin is able to solubilize non-fibrillar parts of collagen, such as telopeptides, while the triple helix structure is less susceptible to non-specific proteases and specifically digested by collagenase. Independently of methodological differences, collagen represents a remarkable part of the insoluble component of *C. andromeda* jellyfish. Several studies focused on peptides and jellyfish hydrolyzed-collagen which have shown a wide range of biological effects, such as immune-modulating and antioxidant activity [48,50,82]. It is not yet clear and poorly studied whether the major bioactivity resides in the non-collagen type hydrolysable with pepsin or in the fibrillar collagen digestible with collagenase. Consequently, the collagen quantification in different JF species and the enzymatic systems able of producing bioactive peptides becomes key information for future research in this field.

##### Total Content of Phenolic Compounds in Pepsin- and Collagenase-Hydrolyzed Fractions

The evaluation of total phenolic compounds (by the Folin–Ciocalteu assay) in the pepsin-hydrolyzed fractions from all jellyfish samples indicated a notable quantity of phenolic compounds, measured as amount of gallic acid equivalent (GAE) per g of dry weight found (Figure 6C). Comparably, (i) 2285 ± 300 µg and 2738 ± 565 µg of GAE/g DW were found in pepsin-hydrolyzed fractions from UMB and OA samples, respectively, after 80% EtOH followed by PBS extractions; (ii) 3256 ± 420 µg of GAE/g of DW were found in UMB samples subjected to PBS followed by 80% EtOH extractions; and (iii) 4838 ± 639 µg of GAE/g of DW was found in the OA fraction after 80% EtOH followed by PBS extractions (Figure 6C). 

Data of phenolic content estimated in collagenase-hydrolyzed samples (Figure 6D) shows a lower quantity of phenolic compounds as compared to the fraction related to the pepsin-hydrolyzed proteins. The quantity of total phenolic compounds was found to be 1103 and 1132 µg GAE/g of DW in the UMB and in the OA sample, respectively, previously subjected to ethanol followed by PBS extractions. Similar values (950 and 1197 µg GAE/g of DW) were found in the samples of UMB and OA subjected to extraction with PBS followed by ethanol extractions. 

In both enzymatic systems, the statistical analysis showed no differences in phenolic content neither between the JF body parts nor between the type of extraction of soluble compounds previously performed, confirming that the pre-treatments did not affect also the phenolic compounds’ content of the insoluble compounds of *C. andromeda*. The presence of phenolic compounds is usually an index of strong antioxidant and chemo-preventive activities related to several protective properties [83,84,85,86]. They are usually found in great amounts into plants, vegetables, and other terrestrial and marine organisms like macro and microalgae and, in this case their presence in jellyfish is likely due to the well-known symbiosis with the microalgae of the family *Symbiodiniaceae*.

##### Antioxidant Activity in Pepsin- and Collagenase-Hydrolyzed Fractions

Surprisingly, high antioxidant activity was also detected in the pepsin-hydrolyzed fractions of the insoluble component of *C. andromeda*, both UMB and OA (Figure 6E). Values of 23,777 nmol TE and 27,903 nmol TE/g of DW were found in UMB and OA, respectively, of jellyfish samples previously extracted with 80% EtOH followed by PBS extraction sequence, the antioxidant activity found in samples of UMB and OA previously extracted with the PBS followed by 80% EtOH sequence, were 34,833 nmol TE and 46,896 nmol TE/mg of proteins; these values were significantly higher than samples treated with 80% EtOH followed by PBS extraction sequence (Figure 6E).

As shown in the Figure 6F, the antioxidant activity measured in all the samples of hydrolyzed collagen derived from both oral arms and umbrella, as well as from both types of pre-extraction, showed a lower antioxidant activity than that measured in pepsin-hydrolyzed samples. 

The detected antioxidant activity was similar in samples of umbrellas and oral arms and no differences were found between samples subjected to the two kinds of extraction. Indeed, the values of antioxidant activity were 8715 nmol TE and 9219 nmol TE/g of DW in UMB and OA of *C. andromeda*, respectively in samples pre-extracted with ethanol-PBS sequence, and values of 8023 nmol and 10,005 nmol TE/g of DW in UMB and OA, respectively in jellyfish samples treated with the PBS followed by 80% EtOH extraction sequence (Figure 6F).

This type of study is preparatory to establish the presence and identify the biochemical components with nutritional, nutraceutical and/or pharmaceutical properties potentially contained in the biomass of jellyfish. Furthermore, this experimental approach sets the investigation methodologies, in order to standardize the procedures necessary for the qualitative and quantitative evaluation of the biochemical characteristics of these organisms. Finally, standardized analysis and established methodologies can be used to search for species from which medicinal drugs, biochemicals, and other material of commercial value can be obtained and set up an efficient methodology for their sustainable exploitation. The quantity and quality of jellyfish nutritional components are clearly influenced by genetic and environmental factors. Usually, proteins are the major jellyfish components, except in jellyfish associated with symbiotic dinoflagellates, where lipids represent a quantitatively and qualitatively important component [21,44,87]. Protein hydrolysates are known to exert a wide spectrum of biological functions including anti-proliferative, anti-cancer, anti-hypertensive, hypocholesterolemic, anti-inflammatory, and antioxidant [88,89,90,91,92,93], and most biological activities are mainly attributed to peptides [94]. Due to their well accepted antioxidant effects, often linked to prevention or reduction of oxidative stress associated to some diseases [95,96,97,98,99], natural peptides, especially those of marine origin can be regarded as a valuable replacement for the synthetic antioxidants [100,101], also because natural peptides penetrate the cells more easily due to the amino acids sequence, composition, and the low molecular weight [102,103,104]. 

## 3. Materials and Methods

### 3.1. Chemicals, Materials, and Equipment

Ethanol and acetonitrile; Bovine Serum Albumin (BSA); ABTS [2,20-Azinobis (3-ethylben-zothiazoline-6-sulfonic acid) diammonium salt]; 3,4,5-Trihydroxybenzoic acid (Gallic acid); phosphate buffered saline (PBS), Potassium persulfate (Potassium peroxydisulfate); (±)-6-Hydroxy-2,5,7,8-tetramethylchromane-2-carboxylic acid (TROLOX); Folin & Ciocalteu’s phenol reagent; pepsin from porcine gastric mucosa (≥2500 U/mg), collagenase from *Clostridium histolyticum*, hydrochloric acid (HCl), 2-mercaptoethanol, sodium carbonate (Na_2_CO_3_), copper (II) sulphate pentahydrate (CuSO_4_ •5H_2_O), and sodium hydroxide (NaOH) were purchased from Sigma-Aldrich (Merck Life Science srl, Milan, Italy). 96 Well Clear Polystyrene Microplate round-bottom was purchased from Corning® (Corning, NY, USA). Bio-Rad Protein Assay Dye Reagent concentrates was purchased from Bio-Rad Laboratories (Munich, Germany). Infinite M200, quad4 monochromator™ detection system was from Tecan group (Männedorf, Switzerland). Potassium sodium tartrate tetra-hydrate (KNaC_4_H_4_O_6_ • 4H_2_O) was purchased from Millipore (Burlington, MA, USA). All buffers, reagents, amino acid standards and the column for the amino acid analysis were obtained from Pickering laboratories (Mountain View, CA, USA).

### 3.2. Jellyfish Samples

*Cassiopea andromeda* (Forsskål, 1775) jellyfish (9 individuals) were sampled inside the harbor “la Cala” of Palermo (Sicily, Italy) in November–December 2017 at a depth between 0.5 and 2 m. After biometric measurement (weight and UMB diameter) umbrella and oral arms of each specimen were easily separated and immediately frozen in liquid nitrogen and stored at −80 °C until lyophilization. Lyophilized samples were then stored at −20 °C until use.

### 3.3. Amino Acid Analysis

The amino acid profile in lyophilized samples was analyzed by a HPLC system (Agilent Infinity 1260, Agilent Technologies) coupled to an on-line post-column derivatization module (Pinnacle PCX, Pickering laboratories, Mountain View, CA, USA), using ninhydrin (Trione) as a derivatizing reagent and Na+-ion exchange column (4.6 × 110 mm, 5 μm). Eighteen standard amino acids, ammonia and taurine were quantified from standard curves measured with amino acid standards. Prior to the analysis, the samples were hydrolyzed in 6 M HCl containing 0.4% mercaptoethanol for 24 h at 110 °C (HCl hydrolysis). Glutamine and asparagine were converted to glutamic and aspartic acid, respectively. Cysteine (Cys) was quantified as cystin (Cys-Cys). The samples were filtered via micro filter, the pH was adjusted to 2.2 and the samples were further diluted with a citrate buffer (pH 2.2) for the HPLC analysis. 

### 3.4. Aqueous and Hydroalcoholic Extractions of Soluble Compounds

Lyophilized jellyfish samples (oral arms and umbrella) were finely powdered with mortar and pestle and liquid nitrogen, and the resulted dry powder was subjected to aqueous or hydroalcoholic extraction followed by sequential enzymatic digestions (Figure 2 and Figure 5).

Water-soluble compounds were extracted by stirring the lyophilized jellyfish samples with 16 volumes (*w*/*v*) of PBS at pH 7.4, for 2 h at 4 °C, by using rotary tube mixer at 25 rpm, then centrifuged at 9000× *g* for 30 min at 4 °C. Hydroalcoholic-soluble compounds were extracted with 16 volumes (*w*/*v*) of 80% ethanol solution (80% EtOH), for 16 h at 4 °C, by stirring with rotary tube mixer at 25 rpm. Samples were then centrifuged at 9000× *g* for 30 min, at 4 °C, and the supernatant were separated from the insoluble material. Each pellet was subjected to a second extraction with different solvent, namely, the pellet resulting from the extraction with PBS was subjected to extraction with 80% EtOH and the pellet resulting from extraction with 80% EtOH was subjected to extraction with PBS. After supernatants separation, all types of soluble compounds were analyzed for protein and phenolic compounds’ content, and for the antioxidant activity as further described. The final pellets, containing non-extractable compounds, was stored for the following enzymatic hydrolysis.

### 3.5. Sequential Hydrolysis of Insoluble Compounds 

The pellets resulting from the solvent extraction, containing insoluble compounds, were subjected to sequential enzymatic hydrolyses with pepsin followed by collagenase. Briefly, the pellets were suspended in 1 mg/mL of pepsin (enzyme/substrate ratio of 1:50, *w*/*w*) in 0.5 M acetic acid and stirred for 48 h at 4 °C. After digestion, the samples were centrifuged at 9000× *g* for 30 min and the pepsin-hydrolyzed proteins in the supernatant were analyzed for protein and phenolic content, and antioxidant activity. Then, the residual pellet, mainly composed by undigested collagen, was washed two times with bi-distilled water, and subjected to a second enzymatic digestion with 6 mg/mL of bacterial collagenase (enzyme/substrate ratio of 1:50, *w*/*w*) in TES buffer 50 mM, pH 7.4 and 0.36 mM of CaCl_2_, at 37 °C, stirred for 5 h. Finally, the samples were centrifuged at 9000× *g* for 30 min, and the hydrolysates soluble in the supernatant were analyzed for protein and phenolic content, and antioxidant activity. The residual pellet was considered as not-hydrolysable jellyfish material (Figure 5).

### 3.6. Protein Quantification 

Protein concentrations were estimated by Bradford assay [78] using bovine serum albumin (BSA) as standard. The assay was modified and adapted to 96-well microplate (Corning). The amount of collagen peptides was also estimated by Lowry assay, first developed by [80], slightly modified by [82] and adapted to 96-well microplate (Corning). Briefly, 45 μL of reagent A (40 mg of potassium sodium tartrate tetrahydrate and 1 g of sodium carbonate in 10 mL of 0.5 M NaOH) and 5 μL of reagent B (20 mg of potassium sodium tartrate tetrahydrate and 30 mg of copper (II) sulphate pentahydrate in 1 mL of 0.1 M NaOH) were added and mixed with 50 μL of samples and then incubated at 50 °C for 20 min in the dark. Then, 150 μL of Folin–Ciocalteu’s reagent 2N (1:15, *v*/*v*) was added to the samples, mixed and incubated at 40 °C for 20 min, and the absorbance was read at 630 nm. A solution of 1 mg/mL of bovine collagen from calf skin (Sigma-Aldrich, Saint Louis, MS, USA), was used as control. Both methods used the Infinite M200, quad4 monochromator™ detection system (Tecan, Männedorf, Switzerland) to analyze the microplates. All samples and controls were evaluated in triplicate.

Total collagen content was estimated by hydroxyproline analysis. Total hydroxyproline content was determined after acidic hydrolysis and HPLC analysis as described in the Section 3.3. Amino acid analysis: Total collagen content of samples in g/100 g dry weight was estimated as follows:

Total collagen content (%DW) = H × CF(1)

where, H = total hydroxyproline content (g/100 g dry weight); CF = conversion factor. 

The general conversion factor for hydroxyproline to collagen is around 8 (AOAC, 1996), indeed, literature estimates of Hyp in collagen vary between 12–14% and hence the Hyp:collagen ratio varies between 7.14–7.69. In this work, we have chosen a value of 7.5 (13.5% Hyp in collagen) following Colgrave et al [105].

The ratio of total collagen as proportion to total protein content was estimated as follows:

Total collagen content (protein basis) (%) = [Estimated collagen content/Total protein content (amino acid basis)] × 100(2)

where, Estimated collagen content (mg/g) = Hyp content (mg/g) × 7.5).

### 3.7. Quantification of Phenolic Compounds 

The content of phenolic compounds in the extracts was determined by a modified Folin–Ciocalteu colorimetric method [106]. The test solutions containing 50 μL of sample were mixed with 50 μL (1:4) of Folin–Ciocalteu phenol reagent and with 100 μL of NaOH 0.35 M. After 5 min, at room temperature in the dark, the absorbance was spectrophotometrically measured at 720 nm. The calibration curve was plotted versus concentrations of gallic acid ranging from 0 to 40 μg/mL, used as standard. The results were expressed as μg of gallic acid equivalents (GAE) per gram of dry extract. 

### 3.8. In Vitro Antioxidant Activity Assay

The antioxidant activity was assayed by TEAC (Trolox Equivalent Antioxidant Capacity) method [107] based on the scavenging of the blue/green ABTS radical [2,20-azinobis-(3-ethyl-benzotiazolie-6-sulfonic acid)], that is converted into a colorless product. The assay was adapted to 96-well microplate (Corning) for Infinite M200. Appropriate blanks with the relative solvent were run in each assay and a Trolox calibration curve was prepared under the same conditions of the samples. Briefly: 10 µL of each sample was added to 200 µL of ABTS+ solution, were stirred and the absorbance was read at 734 nm after 6 min. The antioxidant activity was expressed as nmol of Trolox equivalents (TE) per dry weight (DW) of mg of contained proteins.

### 3.9. Total Lipid Extraction 

One gram of lyophilized jellyfish sample was subjected to hydroalcoholic extraction with 80%EtOH, as described above, and as reported in Leone et al. (2013). The extract was lyophilized and subjected to a total lipid extraction, as well as 200 mg of lyophilized whole jellyfish (WJ) sample, in order to verify the total lipid content of *C. andromeda*. Briefly, total lipids were extracted as the method of Bligh and Dyer [108], with some modifications: 200 mg of dried sample were mixed with a total of 6 mL of chloroform: methanol (2:1) a solution, then other 6 mL chloroform: methanol (2:1) solution and 3 mL of KCl (0.88%) were added in sequence. Samples were shaken for 15 s and centrifuged at 5100× *g* for 5 min. The lower phase was set aside, and the upper phase was subjected to further extraction with 1 volume of chloroform: methanol (2:1) solution. The lower phase was isolated and added to the first one, and mixed with ¼ volume of methanol: water (1:1) solution. In this case the lower phase was put aside, dried in presence of nitrogen flux, and analyzed for lipid composition. 

### 3.10. Fatty Acid Profiles Analysis

Fatty acid methyl esters (FAME) were obtained using boron tri-fluoride (BF_3_): 1/5 of the total lipid extracts in hexane was saponified at 90 °C for 20 min with 3 mL of 0.5 M KOH in methanol. Methyl-tricosanoate (Sigma_Aldrich) was added before saponification, as internal standard. The fatty acids were methylated by adding 2 mL of BF_3_ in MeOH (14%) as reported in Leone and co-workers [21]. The samples were evaporated under a stream of nitrogen, dissolved in 50 μL of hexane, and 1 μL was analyzed by gas chromatography-mass spectrometry (GC-MS).

GC–MS analyses were performed using an AGILENT 5977E gas chromatograph. Separation of compounds was performed on a VF-WAXms (60 m, 0.25 mm i.d., 0.25 mm film thickness, Agilent). The column temperature was maintained at 160 °C for 1 min, and programmed at 4 °C/min to 240 °C for 30 min. Helium was used as a carrier gas (constant flow rate of 1 mL/min) and the mass spectrometer worked in an electron impact mode with a scan range of 50–700 *m*/*z*. The temperature of MS source and quadrupole were set at 230 °C and 150 °C. Analyses were performed in full-scan mode. Compounds were identified by comparing the retention times of the chromatographic peaks with those of authentic standards (F.A.M.E. Mix C_8_–C_24_) analyzed under the same conditions. MS fragmentation patterns were compared with a mass spectrum database, the National Institute of Standards and Technology (NIST) MS 98 spectral database.

### 3.11. Statistical Analysis

Statistical analyses were performed by Graphpad Prism 6.0. An unpaired Student’s *t*-test was used. Differences were considered statistically significant for values *p* < 0.05.

## 4. Conclusions

The presence in the Mediterranean of jellyfish that yearly form blooms, represents a phenomenon worthy of being studied for its ecological and biological aspects, in order to get insight in the biodiversity dynamics. Besides, an in-depth knowledge of the biochemical composition and biological properties of the compounds present, provides the scientific basis for the aware evaluation of the possibilities of exploiting new marine biomasses.

The careful analysis of the biochemical composition of *Cassiopea andromeda* here carried out revealed a conspicuous quantity of compounds of proteinaceous and non-proteinaceous nature, such as digestible proteins, lipids, and phenolic compounds including compounds with considerable anti-oxidant activity. As compared to other non-zooxanthellates jellyfish, *C. andromeda* is richer of polyunsaturated fatty acids and antioxidant extractable bio-molecules. Moreover, these biomasses, with their rich load of important antioxidant compounds [48] and compounds having related bioactivities as anti-cancer [22] and anti-inflammatory activities, are receiving a growing interest as dietary components. In this view, *Cassiopea andromeda* jellyfish, may represent an available and sustainable biomass if the changing of environmental conditions will allow its constant bloom also in the Mediterranean Sea. Furthermore, its ability to host autotrophic zooxanthellae, make it a more sustainable biomass than other jellyfish, also in consideration of its possible rearing at large scale. *Cassiopea andromeda* jellyfish, deserves further studies to highlight all their unexploited sustainable potential.

## Figures and Tables

**Table 1:** Biometric data of *Cassiopea andromeda* specimens, with mean and standard deviation (SD).

Specimens	Umbrella Diameter (cm)	Fresh Weight (g)	Dry Weight (g)	Yield (% FW)
A1	15.5	245.2	18.6	7.6
A2	14.0	233.5	16.3	7.0
A3	17.5	296.5	22.4	7.6
A4	16.5	288.4	19.8	6.9
A5	14.0	202.5	15.7	7.8
A6	14.5	264.9	19.8	7.5
A7	15.5	210.1	17.0	8.1
A8	13.5	152.4	11.6	7.6
A9	14.0	204.5	15.8	7.7
**Mean**	**15.0**	**233.1**	**17.4**	**7.5**
*SD*	*±1.3*	*±46.1*	*±3.1*	*±0.4*

**Table 2:** Amino acid composition of the whole jellyfish biomass of *Cassiopea andromeda*. Data are mean ± Standard deviation (± SD) of four independent analyses. AA, amino acids; Nd, Not detected, ^e^, essential amino acids.

	*Cassiopea andromeda* Whole Jellyfish		
Amino Acids	Percentage per Dry Weight (% of DW)		Percentage of the Total AA (%)
	Mean	*±SD*	
Alanine (Ala)	0.96	*±0.02*	5.98
Arginine (Arg)	1.02	*±0.02*	6.34
Aspartic acid + Asparagine (Asx)	1.29	*±0.07*	8.07
Cystine (Cys-Cys)	0.28	*±0.07*	1.76
Glutamic acid + Glutamine (Glx)	1.80	*±0.03*	11.26
Glycine (Gly)	1.72	*±0.12*	10.73
Histidine (His) ^e^	0.34	*±0.00*	2.13
Hydroxylysine	Nd	*Nd*	0
Hydroxyproline	0.26	*±0.02*	1.64
Isoleucine (Ile) ^e^	0.56	*±0.00*	3.52
Leucine (Leu) ^e^	0.94	*±0.02*	5.84
Lysine (Lys) ^e^	1.06	*±0.01*	6.62
Methionine (Met) ^e^	0.22	*±0.00*	1.38
Methionine sulfoxide	Nd	*Nd*	0
Phenylalanine (Phe) ^e^	0.73	*±0.02*	4.58
Proline (Pro)	0.97	*±0.02*	6.04
Serine (Ser)	0.81	*±0.00*	5.07
Taurine	0.96	*±0.02*	6.01
Threonine (Thr) ^e^	0.53	*±0.10*	3.32
Tryptophan (Trp) ^e^	Nd	*Nd*	0
Tyrosine (Tyr)	0.44	*±0.04*	2.74
Valine (Val) ^e^	0.79	*±0.00*	4.91
Ammonia	0.33	*±0.01*	2.06
Total (AA only)	15.68	*±0.09*	
Total (AA + ammonia)	16.01	*±0.10*	100

**Table 3:** Yield of extraction with aqueous solution (PBS) and hydroalcoholic solvent (80% EtOH); the extracts were lyophilized and compared to the dried whole jellyfish (WJ) samples of *Cassiopea andromeda*. (*) The yield respect to fresh weight (% FW) was theoretically calculated. Data are expressed as mean ± Standard Deviation, SD (*n* = 6).

	Whole Jellyfish (WJ)			
Extraction Solvents	WJ DW (g)	Extract DW (g)	Yield (%DW)	Yield * (% FW)
	Mean *± SD*			
**PBS**	1.629 ± 0.015	1.553 ± 0.011	83.4 ± 15.8	7.1 *
**80% EtOH**	1.517 ± 0.051	0.650 ± 0.009	42.5 ± 0.9	3.2 *

**Table 4:** Content of total lipids extractd by cloroform/methanol from the whole jellyfish and the hydroalcoholic soluble compounds extracted by 80% EtOH from *Cassiopea andromeda* jellyfish. Data are mean of three independent experiments and are expressed as mg/g or as percentage. DW, dry weight; FW, fresh weight; WJ, whole jellyfish; * values theoretically calculated from the average ratio in Table 1.

Total Lipids in *Cassiopea andromeda*		
Total Lipids	Whole Jellyfish (WJ)	Hydroalcoholic Extract (80% EtOH Extract)
Total lipids by DW (mg/g of lyophylized whole JF)	9.4 ± 0.4	6.2 ± 0.5
Percentage of DW (%)	0.94%	0.62%
Lipids in hydroalcoholic extract (mg/g of 80% EtOH)	-	15.5 ± 0.5
Total lipids by FW (mg/g of FW) *	0.67 ± 0.07	0.46 ± 0.09
Percentage of FW * (%)	0.07%	0.05%

**Table 5:** Content and composition of fatty acids in whole jellyfish (WJ) and hydroalcoholic soluble compounds extracted by 80% ethanol from *Cassiopea andromeda* jellyfish. Data are mean of three independent experiments and are expressed as percentage of the total fatty acids.

*Cassiopea andromeda* Fatty Acid Composition		
Fatty Acid (FA)	Whole Jellyfish (WJ)	HydroalcoholicExtract (80% EtOH Extract)
	%	%
	*Saturated FA (SFA) %*	
Lauric acid *C12:0*	9.3 ± 0.9	1.5 ± 0.2
Myristic acid *C14:0*	4.2 ± 0.4	5.2 ± 0.5
Palmitic acid *C16:0*	21.9 ± 2.2	13.9 ± 1.4
Stearic acid *C18:0*	12.5 ± 1.2	9.9 ± 0.9
Arachidic acid *C20:0*	0.6 ± 0.1	0.6 ± 0.1
**Total SFA**	**48.5 ± 4.8**	**31.1 ± 3.1**
	*Monounsaturated FA (MUFA) %*	
Palmitoleic acid *C16:1* (ω7)	4.3 ± 0.4	3,3 ± 0.3
Oleic acid *C18:1 cis*-9 (ω9)	2.8 ± 0.3	2.3 ± 0.3
Isoleic acid *C18:1 trans-10*	0.5 ± 0.1	0.6 ± 0.1
**Total MUFA**	**7.5 ± 0.8**	**6.1 ± 0.6**
	*Polyunsaturated FA (PUFA) %*	
Linoleic acid *C18:2 cis-9,12* (ω6)	0.8 ± 0.1	1.9 ± 0.2
Isolinoleic acid *C18:2 cis-6,9* (ω9)	0.5 ± 0.1	--
Linolenic acid *C18:3 cis-9,12,15* (ω3)	2.6 ± 0.3	3.2 ± 0.3
Stearidonic acid *C18:4* (ω3)	7.4 ± 0.7	7.9 ± 0.8
Arachidonic acid *C20:4* (ω6)	14.2 ± 1.4	19.2 ± 1.9
Eicosapentaenoic acid *C20:5* (ω3)	2.1 ± 0.2	3.5 ± 0.3
Docosatetraenoic acid *C22:4* (ω6)	2.9 ± 0.3	4.2 ± 0.4
Docosapentaenoic acid *C22:5* (ω3)	2.5 ± 0.2	5.1 ± 0.5
Docosahexaenoic acid *C22:6* (ω3)	11.0 ± 1.1	17.8 ± 1.8
**Total PUFA**	**44.0 ± 4.4**	**62.8 ± 6.3**
**Total fatty acids (%)**	**100.0**	**100.0**
Fatty acids *Σω6*	17.9	25.3
Fatty acids *Σω3*	25.6	37.5
**Ratio *ω*6/*ω*3**	**0.7**	**0.7**

**Table 6:** Yield of extractions with aqueous solution (PBS) and hydroalcoholic solution (80% EtOH) compared to the lyophilized umbrella (UMB) and Oral Arms (OA) samples of *Cassiopea andromeda*. Data are expressed as mean (*n* = 6) ± Standard Deviation, SD.

	Umbrella (UMB)			Oral Arms (OA)		
Extraction Solutions	UMB DW (g)	Extract DW (g)	Yield (%DW)	OA DW (g)	Extract DW (g)	Yield (%DW)
	Mean *±SD*				Mean *±SD*	
**PBS**	0.652 *± 0.155*	0.616 *± 0.10*	94.5 *± 0.92*	0.978 *± 0.15*	0.937 *± 0.01*	95.8 *± 0.81*
**80% EtOH**	0.515 *± 0.172*	0.214 *± 0.07*	41.6 *± 0.09*	1.003 *± 0.08*	0.436 *± 0.04*	43.5 *± 0.45*
